# Slow Breathing and Hypoxic Challenge: Cardiorespiratory Consequences and Their Central Neural Substrates

**DOI:** 10.1371/journal.pone.0127082

**Published:** 2015-05-14

**Authors:** Hugo D. Critchley, Alessia Nicotra, Patrizia A. Chiesa, Yoko Nagai, Marcus A. Gray, Ludovico Minati, Luciano Bernardi

**Affiliations:** 1 Brighton and Sussex Medical School, University of Sussex, Brighton, United Kingdom; 2 Sackler Centre for Consciousness Science, University of Sussex, Brighton, United Kingdom; 3 Imperial College Healthcare NHS Trust, London, United Kingdom; 4 Department of Psychology, Sapienza University of Rome, Rome, Italy; 5 Ghermann Laboratory, University of Queensland, Queensland, Australia; 6 IRCCS, Fondazione Santa Lucia, Rome, Italy; 7 IRCCS, Fondazione Neurologico Carlo Besta, Milano, Italy; 8 Department of Internal Medicine, University of Pavia and Fondazione IRCCS Policlinico San Matteo, Pavia, Italy

## Abstract

Controlled slow breathing (at 6/min, a rate frequently adopted during yoga practice) can benefit cardiovascular function, including responses to hypoxia. We tested the neural substrates of cardiorespiratory control in humans during volitional controlled breathing and hypoxic challenge using functional magnetic resonance imaging (fMRI). Twenty healthy volunteers were scanned during paced (slow and normal rate) breathing and during spontaneous breathing of normoxic and hypoxic (13% inspired O_2_) air. Cardiovascular and respiratory measures were acquired concurrently, including beat-to-beat blood pressure from a subset of participants (N = 7). Slow breathing was associated with increased tidal ventilatory volume. Induced hypoxia raised heart rate and suppressed heart rate variability. Within the brain, slow breathing activated dorsal pons, periaqueductal grey matter, cerebellum, hypothalamus, thalamus and lateral and anterior insular cortices. Blocks of hypoxia activated mid pons, bilateral amygdalae, anterior insular and occipitotemporal cortices. Interaction between slow breathing and hypoxia was expressed in ventral striatal and frontal polar activity. Across conditions, within brainstem, dorsal medullary and pontine activity correlated with tidal volume and inversely with heart rate. Activity in rostroventral medulla correlated with beat-to-beat blood pressure and heart rate variability. Widespread insula and striatal activity tracked decreases in heart rate, while subregions of insular cortex correlated with momentary increases in tidal volume. Our findings define slow breathing effects on central and cardiovascular responses to hypoxic challenge. They highlight the recruitment of discrete brainstem nuclei to cardiorespiratory control, and the engagement of corticostriatal circuitry in support of physiological responses that accompany breathing regulation during hypoxic challenge.

## Introduction

The respiratory system exerts a potent influence on the autonomic nervous coordination of bodily processes. One route through which respiration modulates cardiovascular function is via the baroreflex. Arterial baroreceptors are activated by pressured ejection of blood from the heart. Baroreceptor signals are relayed to the medulla to gate reflexively autonomic outflow: Muscle sympathetic nerve activity constricts vascular beds to increase blood pressure, while parasympathetic activity, via the vagus nerve, slows heart rate to help decrease blood pressure. Psychological, physical and chemosensory stress suppresses the baroreflex, permitting blood pressure and heart rate to rise together; an effect which, if exaggerated or sustained, is detrimental to cardiovascular health [1].

Interestingly, breathing at a slow respiratory rate of six breaths per minute can evoke effects that are positively beneficial to cardiovascular health: Slow breathing enhances baroreflex sensitivity in healthy individuals and in patients with heart failure [2] [3]. Slow breathing also reduces muscle sympathetic nerve activity, attenuating hypertensive vasoconstriction [4], and blocks cardiovascular responses to physiological stress evoked by mild hypoxia [5] [2]. Thus, health benefits might result from training people to breathe at approximately half the average resting rate. Yoga practitioners represent one section of society that engages in slow breathing exercises for both psychological and physical health benefits. Mental states of calmness and wellbeing reportedly accompany slow yogic breathing and associated physical positions and manoeuvres. However, these effects are not exclusive to practices from eastern religious traditions [6]. The voluntary modulation of breathing rate therefore offers a channel through which autonomic activity can be shaped to improve both cardiovascular and psychological health.

Animal studies of respiratory and cardiovascular control emphasize brainstem mechanisms [7]. Oscillatory drivers to respiratory rhythm are identified within the retrotrapezoid/parafacial respiratory region and preBötzinger complex [8] [9]. Functional neuroimaging in humans extends this knowledge to identify homologues of such brainstem respiratory centres within pons and medulla [10–13]. Importantly, human neuroimaging highlights the contribution of forebrain centres to reflexive and volitional regulation of breathing and representation of respiratory sensations [11] [14–18]. Neuroimaging is also increasing our understanding of interactions between visceral state (including respiration) and cognition or emotion [19]. However, the neural mechanisms through which slow breathing impacts on both mental and physical states remain poorly understood.

The present study was motivated to detail central control of breathing and associated autonomically-mediated cardiovascular responses to hypoxia in humans under conditions of normal rate and slow breathing. Specifically, we sought to identify the neural mechanisms underlying a hypothesized attenuation by slow breathing of cardiovascular ‘stress’ responses to hypoxia. In achieving this aim, we examined the neural, respiratory and cardiovascular responses to hypoxic challenge at different breathing rates and identified neural substrates associated with generating and representing the evoked physiological changes.

## Materials and Methods

### Participants

Twenty-three healthy volunteer participants (8 women 12 men; age S.D. = 34.5 ± 10.4 yrs) were recruited by advertisement to the study, which was approved by the Brighton and Sussex Medical School Research Governance and Ethics Committee (BSMSRGEC), University of Sussex (approval number 10/033CRI). Each participant gave full informed consent in writing, in accordance with procedures approved by BSMSRGEC. Data from twenty participants were included in final analyses since insufficient /incomplete datasets were acquired in the remaining three participants. Seven of the participants could be classified as ‘trained breathers’ on the basis of regular yoga practice, accomplished wind instrument playing or regular recreational or professional diving.

### Experimental task and hypoxic challenge

During scanning, the participant lay supine on the scanner bed and wore a respiratory mask fitting over nose and mouth which was attached to breathing circuit, through which she/he breathed atmospheric air alternated with hypoxic gas mixture (13%O_2_). The circuit incorporated a pneumotachograph to permit precise measurement of ventilatory volumes, and was attached via polythene tubing to a capnograph. The participant also viewed a projection screen via a mirror mounted on the head coil and wore electrostatic headphones for delivery of auditory cues and attenuation of scanner noise. The participant held a button box, though which she/he could make ratings at specific times during the experiment.

The main experimental session was undertaken over a continuous period (26.5 minutes-27.5 minutes, depending on pseudorandomised order, see below), during which time the participant was prompted, over blocks of one minute either to breath at their own pace (unpaced), to breath slowly (slow paced), or to breath more rapidly (regular paced—approximating the normal supine breathing rate of most participants). Instructions were given as auditory cues at the onset of each block and throughout the paced breathing at the target rate (‘breathe in’, ‘breathe out’). The participant also heard timing tones every second throughout each block. The participant was prompted visually at the start of each block to shut her/his eyes, and auditorily at the end of each block to open her/his eyes. Between experimental blocks, the participant completed two visual analogue ratings of how alert and how of positive/negative she/he felt at that time. For alertness, the prompt word ‘ALERT’ was written above a horizontal linear scales from -50 (very sleepy) to +50 (maximally awake). For emotional feeling state, the word ‘FEELING’ was presented above a similar scale where -50 indicated very bad and +50 very good. Each participant practiced manipulating the fine position of a cursor along the scale using left and right button presses before the experiment.

**Fig 1:**
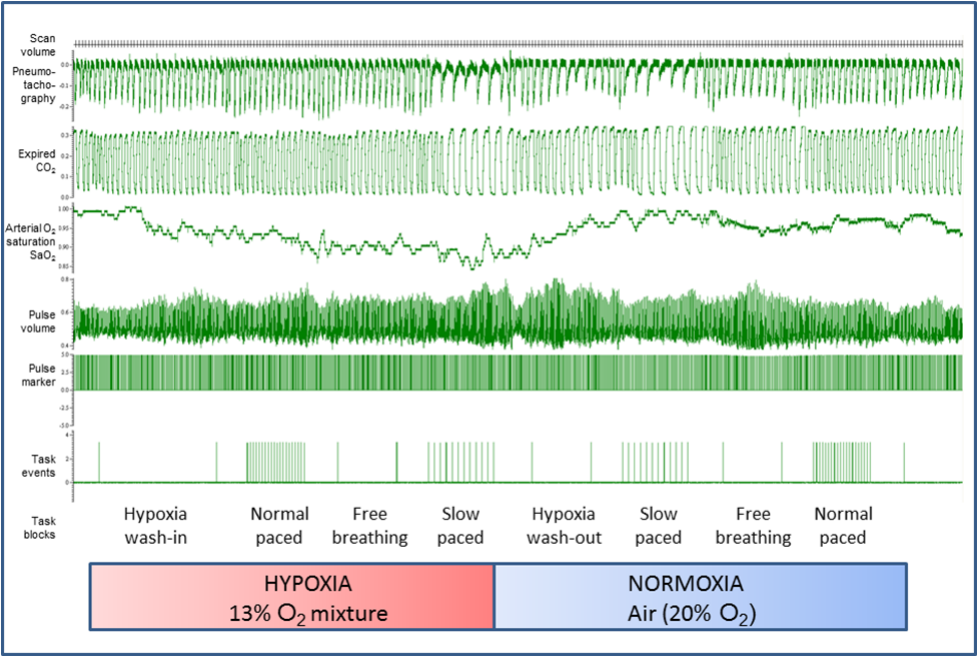
Physiological data acquisition, illustrating experimental design. The figure shows data recording (CED Spike software, Cambridge) during the experiment in which participants breathed normoxic and hypoxic air mixtures over periods of approximately 5 minutes at their natural spontaneous rate (unpaced) and at paced rates approximating to a regular rate (paced normal rate: 9.9 breaths per minute) and at a slow rate (paced slow rate 5.5 breaths per minute). These task conditions were counterbalanced within and between participants and for hypoxia periods were preceded by a 1.5 minute ‘wash-in’ period and followed by a 1 minute ‘wash-out’ period. During the experiment, volume changes associated with breathing were recorded directly using pneumotachography alongside measurement of expired CO_2,_ and through pulse oximetry, arterial oxygen saturation, pulse volume and pulse rate. The experiment entailed repetitions of task conditions in hypoxia and normoxia. For a subset of participants, beat-to-beat blood pressure was also recorded.

During the experimental session, the gas breathed by the participant was switched between atmospheric (normoxic) air and a hypoxic mixture. Twelve of the participants began with a period of normoxic breathing, undertaking three one minute blocks (unpaced, slow paced and regular paced breathing). They were then switched to the hypoxic mixture. After a 1.5 minute ‘wash-in’ block, they performed the three one-minute blocks (unpaced slow paced, regular paced), followed by a 1 minute ‘wash-out’ block breathing atmospheric air. The sequence of normoxic / hypoxic periods was then repeated. The remaining eleven participants began the experimental session breathing the hypoxic mixture, beginning with the 1.5 minute ‘wash-in’ period performing the three blocks in hypoxia followed by the ‘wash-out’ period. Pseudorandomization of the unpaced, slow paced and regular paced blocks within each normoxic/hypoxic periods and across participants ensured control of order effects. During hypoxia wash-in and wash-out blocks, the participant was prompted to breathe at own pace with closed eyes. The experimental session (including presentation of cues and recording of ratings) was controlled through a programme running in Matlab (MathWorks, Natick) and synchronised with scan acquisition (Fig 1).

### Physiological monitoring and analyses

During scanning, the participant’s respiration was monitored using pneumotachography (calibrated at the end of each session) for ventilatory volume (Bernardi et al 2006) and capnography for tidal CO2 level (Oridion Microcap) along with oxygen saturation from pulse oximetry (Nonin 8600FO). Cardiovascular data was recorded during scanning also using pulse oximetry for heart timing and an index of pulse amplitude. In seven of the participants, beat-to-beat blood pressure data was also acquired using a Portapres (Finapres Medical Systems BV) that had been re-engineered in-house to be MR compatible. In these participants, the blood pressure sensor was placed on the left index finger and pulse-oximeter finger stage on left ring finger; in all others the pulse oximeter was on the left index finger. All physiological signals were recorded, along with timing signals indicating the timing of scans (individual echoplanar image volumes) and of the different experimental blocks, via an analogue-digital converter (CED1401) into Spike Software (Cambridge Electronic Design, UK) (Fig 1). Data were subsequently exported into Matlab, cleaned of artefact and averaged for statistical comparisons of block effects. They were also resampled to enter as regressors into brain imaging analyses for interpretation of regional brain activity.

### Neuroimaging data acquisition and analysis

The research was conducted at the Clinical Imaging Sciences Centre, Brighton and Sussex Medical School (CISC-BSMS) on a 1.5 Tesla Siemens Avanto Magnetic Resonance scanner. To obtain time-series datasets indexing haemodynamic correlates of changes in regional brain activity, we acquired continuous T2* echoplanar images (EPI) sensitive to blood-oxygenation level dependent (BOLD) contrast while each participant underwent the experimental session (isotropic 36 x 3mm interleaved slices, TE/TA = 50/3208ms, 30° tilt to A-P commissural plane). Pre-processing of neuroimaging datasets and subsequent neuroimaging analyses were undertaken using Statistical Parametric Mapping software (SPM8; http://www.fil.ion.ucl.ac.uk/spm/) on a Matlab computational platform. Functional imaging datasets (36 slice EPI volumes) were pre-processed with correction for slice timing, between-volume movement, spatial normalization to MNI standard space and smoothing with a Gaussian kernel FWHM 8mm to give 2mm^3^ resampled voxel size.

For each participant, time-series neuroimaging data were entered into two separate sets of first-level analyses: 1) Task blocks of interest (unpaced, slow paced and regular paced breathing in normoxia and hypoxia) were modelled alongside blocks of no interest (hypoxic wash-in and wash-out periods) and potentially confounding physiological covariates (changes in CO_2_, SaO_2_) to test for effects of experimental manipulations 2) Individual physiological measures, changing over the course of the experimental session, were convolved with a canonical haemodynamic response function (controlling for haemodynamic lag from putative underlying neural activity) and down-sampled to the frequency of neuroimaging data acquisition to permit identification through scan-by-scan regression analyses of regional brain activity fluctuating with end-tidal CO_2_, arterial oxygen saturation SaO_2_, respiratory rate, tidal and minute volume (ventilation), heart rate and standard deviation of interbeat interval. These analyses were therefore tuned to brain responses related to physiological changes occurring at a temporal resolution of 3.21s and above, providing fine-grained information about the central control of peripheral autonomic responses and their relationship to, including interactions with experimental task manipulations.

In both sets of analyses, six movement regressors were also included as confounding covariates. Data were filtered in the time domain using a high-pass filter (cut-off period of 128 seconds), removing signal drift and increasing sensitivity to specific conditions, associated physiological changes and their interaction. We also adjusted for global signal using proportional scaling. Second-level analyses were conducted to test; 1) within an ANOVA modelling the task conditions, for main effects and interactions of the experimental manipulations, and 2) using one-sample T-tests, for consistent effects of physiological changes on regional brain activity across the group. Effects are reported at a significance of P<0.05 corrected, determined using the combination of using at a voxel-wise threshold significance of P<0.001 uncorrected in combination with a cluster extent threshold > 41 contiguous voxels (computed through Mont Carlo simulation with 1000 iterations [20]. In addition, when reporting activity within small ventral striatal and brainstem nuclei and functional neural correlates of beat-to-beat blood pressure change (acquired in a subset of participants) data are described at the same voxel-wise threshold, omitting the cluster extent threshold.

## Results

### A) Subjective experience

At the start of the study, and after each task condition, participants rated their subjective degree of alertness and comfort on a visual analogue scale controlled by the button-box (see Methods). Across all participants and task conditions, there was no overall effect of task condition on reported comfort or alertness at criterion significance (there was only a trend for a hypoxia x breathing rate interaction for alertness (F(2,34) = 2.61 p = 0.09) condition, toward increased alertness during slow vs normal rate breathing during normoxia but not hypoxic conditions). At debriefing, the majority of the participants reported the experimental setup with breathing apparatus and associated monitoring equipment to be uncomfortable. Overall, this discomfort only slightly diminished over the course of the experiment, and there was accompanying trend in the attenuation of scores of alertness (for first vs fourth quarter of the experiment; mean rated comfort: 28.5.0 vs 29.0; mean rated alertness: 30.2 vs 29.2. Effect of task order across experiment for comfort (F1, 19) = 1.6, n.s. for alertness; F(1, 19) = 0.85, n.s.). The experimental conditions were pseudo-randomized and counterbalanced across participants and it is likely that non-task related discomfort overshadowed influences of mild hypoxia and respiratory rate on rated subjective wellbeing and alertness.

### B) Physiological changes evoked by task conditions

**Fig 2:**
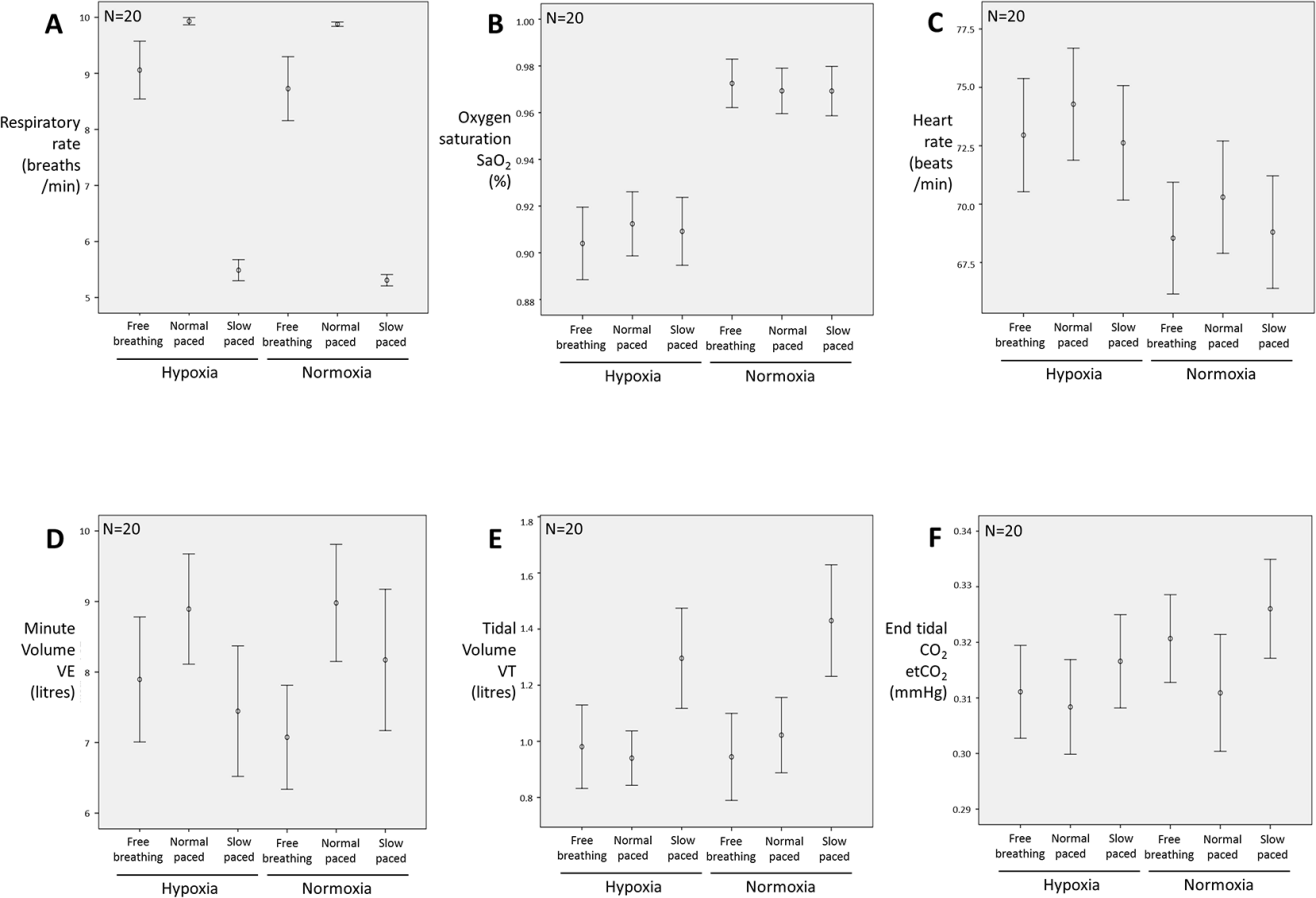
Group physiological effects elicited by the experimental manipulations. Error plots of average physiological response measured in the experimental different task conditions. The data validate the experimental manipulations for respiratory rate and oxygen saturation (Fig 2A and 2B) and show a significant effect of hypoxia on heart rate (Fig 2C). Task effects were also observed on minute ventilation (VE; Fig 2D), tidal volume (VT; Fig 2E) and end tidal CO_2_ level (Fig 2F). Results for other parameters are presented within the text.

Over the course of the study, controlled breathing rates closely matched the pacing cues under both hypoxia and normoxia (paced slow rate breaths/min: hypoxia 5.5±0.84, normoxia 5.3±0.45; paced normal rate hypoxia 9.9±0.29, normoxia 9.9±1.7). The spontaneous (unpaced) breathing rates were close to the paced normal breathing rate, though averaged lower on account of three individuals who had relatively slow breathing rates (below 8 breaths per minute) and who were among the group of seven ‘trained breathers’. The trained breathers, compared to the other participants, did not show any systematic differences in physiological reactivity to the task conditions. There was no significant difference in spontaneous unpaced) breathing rates between hypoxic (9.1±2.3) and normoxic (8.7±2.5) conditions (Fig 2A)

Hypoxic challenge, i.e. breathing the 13% O_2_ in nitrogen gas mixture, compared to normoxia, evoked a reduction in arterial oxygen saturation (SaO2) from 97.0% to 90.8% on average across the group. This main effect of hypoxia was significant (F(1,19) = 54.7, p<0.001). There were no significant differences in the magnitude of this effect between the different breathing conditions (Fig 2B). Hypoxia was associated with a general increase in heart rate across the different breathing conditions (mean beats per minute ± S.D.: normoxia 69.2±10.6; hypoxia 73.3±10.7 F(1,19) = 36.5, p<0.001) (Fig 2C. Hypoxia evoked no consistent differences in heart rate variability (standard deviation of pulse intervals, s^-1^: hypoxia 0.058±0.02; normoxia 0.062±0.02; n.s).

The minute volume (VE) differed across breathing rate levels (F(2,38) = 13.4, p<0.001), being greater for normal rate paced breathing than slow paced or spontaneous conditions. There was also a significant interaction for VE between breathing rate and hypoxia (F(2,38) = 5.0, p = 0.01), whereby the difference in VE between fast and slow breathing was significantly larger under hypoxia (8.9±3.5 vs. 7.4±4.1 min^-1^, p_Bonf_<0.001) but not under normoxia (9.0±3.7 vs. 8.2±4.5 min^-1^) (Fig 2D). Tidal volume (VT) was greatest for the slow paced conditions (F(2,38) = 13.2, p<0.001), but unaffected by hypoxic challenge (Fig 2E).

End tidal CO_2_ (etCO_2_) decreased on average under hypoxia (31.2±3.7 vs. 31.9±4.1 mmHg, F(1,19) = 8.7, p = 0.008) and changed with breathing rate (F(2,38) = 8.0, p = 0.001), being lower for fast compared to slow breathing when averaged across hypoxic and normoxic conditions (31.0±4.2 vs. 32.1±3.8 mmHg, p_Bonf_<0.001). There was however no significant interaction between hypoxia and breathing rate (Fig 2F).

**Fig 3:**
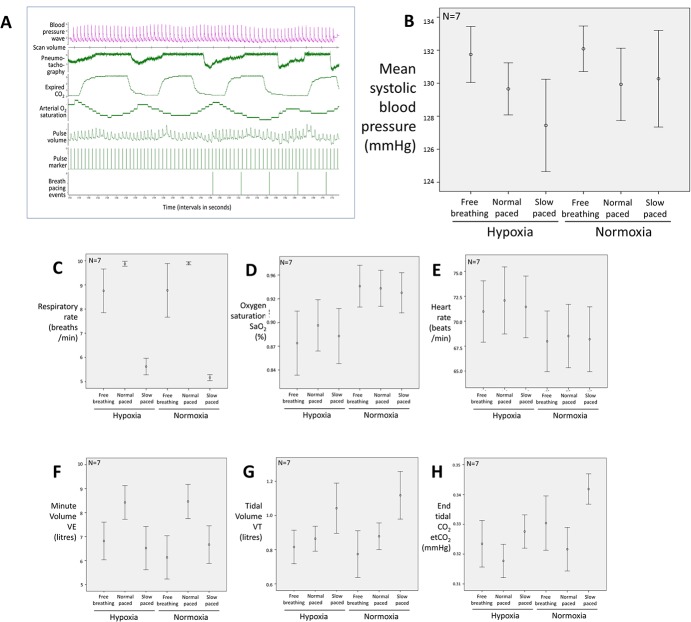
Physiological data including beat-to-beat blood pressure in subset N = 7. **A)** Beat-to-beat blood pressure acquisition during fMRI. The figure presents a close-up snapshot of physiological data recording as in Fig 1, but also including recording of beat-to-beat blood pressure, which was acquired in the experiment from 7 of the 20 participants. **B)** Beat-to-beat blood pressure effects elicited by experimental manipulations. The figure mean data for the blood pressure changes recorded from across the subset of participants (N = 7). Observed differences were non-significant at standard statistical threshold. **C)-H)** Experimental evoked physiological responses for the subset of participants). The figure shows the pattern of physiological changes in the subset of participants in whom blood pressure was recorded for comparison with the whole group data depicted in Fig 2. The responses within this subset closely mirrored those seen for the whole group (N = 20).

Beat-to-beat blood pressure was recorded concurrently with fMRI in a subset of participants (N = 7, including one ‘trained breather’) (Fig 3). Blood pressure differed non-significantly across task conditions (F(1,6) = 0.17, n.s.) (Fig 3B). The physiological effects of task (SaO2, breathing rates, HR, VT, VE, HR and HRV) apparent across the larger (N = 20) group were mirrored across this subset of participants, indicating that they were representative of the whole group (Fig 3C–3H). There was a trend in the relationship between heart rate and blood pressure that was consistent with baroreflex suppression by hypoxia.

### C) Neuroimaging changes associated with task conditions

In the course of exploratory neuroimaging analyses, we observed no supra-threshold differences between ‘trained breathers’ and other study participants in brain responses to task conditions or correlates with measured peripheral physiology. Consequently, we pooled participants into one group. Post hoc assessments of regional neuroimaging findings did not indicate that these regional effects across the group were driven by this subset of participants.

#### Hypoxia versus normoxia

**Fig 4:**
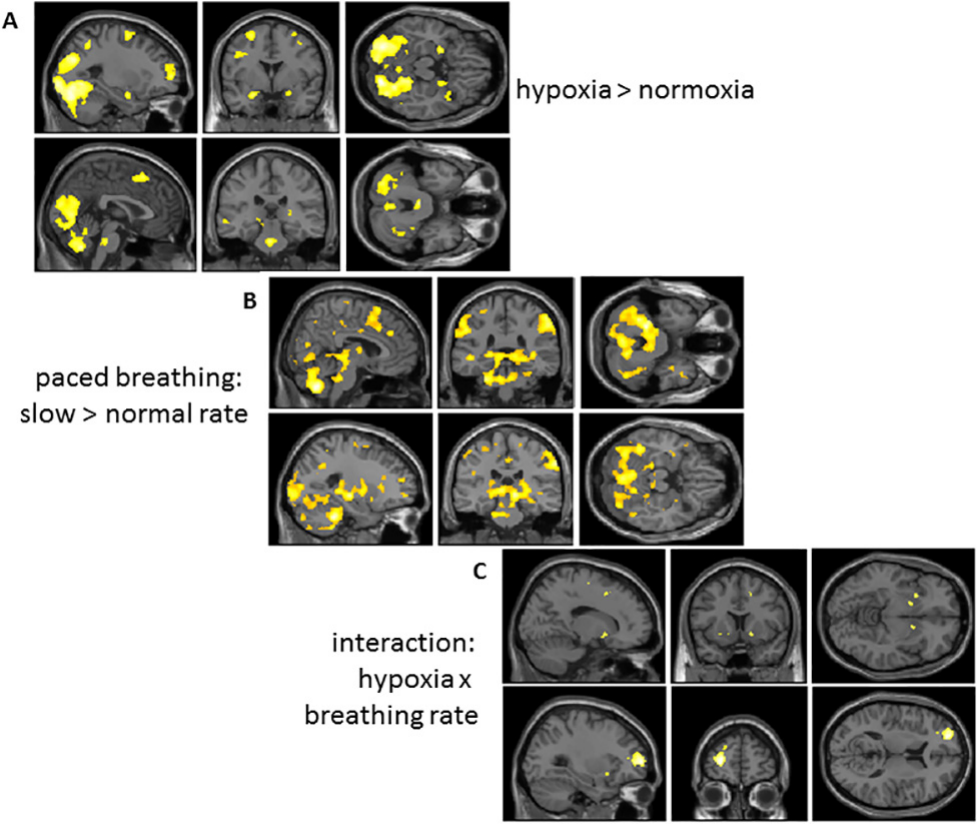
Brain responses to experimental task manipulations. Group data is presented on sagittal coronal and horizontal sections of a normalized template brain to illustrate suprathreshold activity differences associated with task conditions. Data is illustrated at a significance of P<0.05 corrected, determined using the combination of using at a voxel-wise threshold significance of P<0.001 uncorrected in combination with a cluster extent threshold > 41 contiguous voxels (computed through Mont Carlo simulation with 1000 iterations [20]). **A)** Main effect of hypoxia: increased activity when breathing 13%O_2_ gas mixture v. normoxic air. Increased activity during hypoxic challenge is observed within regions including occipitotemporal cortex, amygdala and pons. **B)** Main effect of paced breathing rate: increased activity associated with paced slow breathing v. paced normal rate breathing: Increased activity during slow relative to normal rate breathing is observed within regions including cerebellum, sensorimotor cortices, dorsal pons, midbrain and thalamus. **C)** Activity reflecting interaction between presence and absence of hypoxia during paced normal rate v. slow breathing. Activity within left lateral frontal pole and ventral striatum reflects the impact of slow breathing on brain response to hypoxia.

Hypoxic challenge evoked dominant increases in regional brain activity, with noteworthy activation within mid dorsal pons, bilateral amygdala, thalamus and cerebellar cortices. Within neocortices, hypoxia was associated with enhanced activation of occipital and medial and dorsolateral prefrontal regions (Fig 4). There were no clusters of greater activity during normoxia compared to hypoxia (Table 1).

**Table 1:** Regional activation related to task conditions.

Location of cluster peak	side	MNI coordinates of peak	Extent	Peak voxel T-score
**Hypoxia greater than normoxia**				
Ventral occipital and fusiform cortices bilaterally	L	-12,-84,-12	17285	5.20
	R	16,-78,-8		5.15
	R	20,-80,-18		5.09
Brainstem: mid pons	L	-2,26,-30	11	4.40
Dorsomedial prefrontal cortex (supplementary motor area)	L	-2,20,58	514	4.40
	R	8,12,54		4.21
	L	-8,10,56		3.88
Anterior insula	L	-44,22,-4	300	4.25
Lateral frontal pole	L	-28,56,6	398	4.22
Amygdala bilateral	L	-24,0,-20	86	4.17
	R	22,0,-18	55	3.76
Uncus/ anterior insula	R	36,10,-22,	195	4.13
Dorsolateral prefrontal cortex	L	-54,22,20	882	4.08
	R	50,16,30	222	4.07
	R	48,16,48	79	3.84
Posterior superior temporal gyrus	L	-62,-48,-4	259	4.07
Hypothalamus / substantia nigra	L	-8,-12,10	42	4.05
Lateral superior frontal gyrus	L	-26,0,62	324	3.98
Angular gyrus	L	-28,-54,52	117	3.90
Middle frontal gyrus	R	46,22,14	42	3.81
Thalamus	R	14,-12,10	41	3.7
**Normoxia greater than hypoxia**				
No supra threshold clusters	-	-	-	-
**Paced>unpaced breathing**				
Middle frontal gyrus	R	32,34,16	50	3.94
**Unpaced>paced breathing**				
Mid insula	R	50,-4,0	45	3.50
**Slow rate paced breathing more than normal rate paced breathing**				
Cerebellum (extended cluster encompassing dorsal brainstem and striatum)	L	-20,-40,-34	24014	5.94
Midbrain: periaqueductal grey		-8,26,4		4.63
Brainstem: dorsal pons		-6,-30,-30		4.20
Ventral striatum	L	-14,0,-2		4.78
	R	12,4,-4	199	4.23
Thalamus	R	18,-8,4	71	4.16
Dorsal anterior cingulate cortex	R	8,38,32	103	4.12
Temporal pole	R	38,2,-40	133	4.07
Superior frontal gyrus	R	30,2,66	7337	5.25
Superior parietal lobule	L	-54,-36,42	935	4.66
**Normal rate paced breathing more than slow rate paced breathing**				
No supra threshold clusters	-	-	-	-
**Interaction: hypoxia(normal v. slow rate)>normoxia(normal v. slow rate)**				
Lateral frontal pole	L	-26,58,8	350	4.44
Pre supplementary motor area	R	12,20,50	60	4.10
Cerebellum	R	42,-74,-32	45	3.72
Ventral striatum,	L	-16,6,-6	32	3.84
		16,8,-8	14	3.53
**Interaction: hypoxia(slow v. normal rate)>normoxia(slow v. normal rate)**				
No supra threshold clusters	-	-	-	-

#### Controlled (paced) breathing

We tested first for an effect of spontaneous (unpaced) versus paced breathing by contrasting brain activity during controlled (paced) breathing at slow and normal rates. Spontaneous breathing was associated with greater activation of right mid insula cortex, in contrast to controlled breathing which evoked greater activity within right dorsolateral prefrontal cortex. We then contrasted the effects of controlled breathing at slow versus normal rates on regional brain activity. Controlled breathing at the slower rate of 5.5 breaths per minute evoked much greater activity than normal rate paced breathing within brainstem, across the dorsal length of the pons into PAG, in hypothalamic and thalamic regions, within cerebellar vermis and lateral cortices and in regions of striatum and hippocampus and motor, supplementary motor and parietal cortices (Fig 4; Table 1).

#### Interaction between paced breathing rate and hypoxic challenge

Independent of the main effects of hypoxia and paced breathing rate, activation within the left frontal pole and bilateral ventral striatum reflected the interaction of these two task manipulations. The implications of this effect are intriguing as we did not see a significant interaction in either the subjective or physiological responses to the task conditions (Fig 4; Table 1).

### D) Neuroimaging changes associated with physiological measures

#### End tidal CO2

CO2 levels are known to impact on cerebrovascular reactivity and arguably produce global signal changes in functional neuroimaging experiments using BOLD contrast. We tested first for direct voxel wise effects of end tidal CO2 levels first without any global correction, and again using proportional scaling. Both analytic approaches gave essentially the same suprathreshold results. Increasing CO2 levels were associated with enhanced activity within bilateral mid posterior insula cortices. There were no significant cortical or subcortical activations accompanying decreases in fluctuating end tidal CO2 levels (Table 2).

**Table 2:** Regional activation related to physiological regressors.

Location of cluster peak	side	MNI coordinates of peak	Extent	Peak voxel T-score
**CO**_**2**_ **increases**				
Posterior insula – mid insula	L	-50,-26, 22	790	7.95
Dorsal postcentral gyrus	L	-36,-24, 50	230	4.25
Mid-posterior insula	R	44, 0, 2	559	4.17
Ventral posterior insula	L	-38,-22, -2	56	3.94
**CO**_**2**_ **decreases**				
No suprathreshold voxels	-	-	-	-
**Heart rate increases**				
No suprathreshold voxels	-	-	-	-
**Heart rate decreases**				
Premotor cortex (extensive cluster)	L	-60,2,16	46855	8.86
Caudate/putamen	R	22,12,-2		7.51
	L	-30,-16,2		7.15
	L	-22,12,4		7.05
Posterior insula	L	-40,-6,14		5.83
	R	44,-6,10		5.64
Thalamus	R	16,10,0		6.05
Brainstem: dorsal mid pons	R	12,-38,-32		5.38
		-12,-32,-34		4.21
Genual anterior cingulate cortex	L	-2,32,0,	116	4.91
	L	-12,42,4		4.10
	L	-8,38,12		3.84
Superior frontal gyrus	R	24,-6,62	87	4.82
Cerebellum	L	-40,-68,-50	80	4.79
Frontal pole	L	-26,54,2	110	4.78
Anterior hippocampus	L	-30,-6,-32		6.46
Anterior hippocampus / amygdala	R	20,-8,-24	63	4.75
Parahippocampal gyrus	R	36,-6,-36		4.17
Superior temporal gyrus	R	52,-2,-22	87	4.62
Dorsal anterior cingulate cortex	R	6,14,24	84	4.47
**Respiratory rate increases**				
Lateral anterior putamen	R	30,12,-2	41	5.67
Dorsolateral prefrontal cortex middle frontal gyrus	R	36,40,12	44	5.47
Inferolateral prefrontal cortex	R	50,24,14	48	4.46
	R	58,24,24		4.24
**Respiratory rate decreases**				
Periventricular white matter signals	R	24,-24,12	81	4.5
	R	22,-10,28	49	4.42
	L	-30,-40,0	60	4.22
**Heart rate variability increases (sd[pulse])**				
Posterior hippocampus	L	-26,-42,2	338	5.16
White matter clusters	R	24,12,30	147	5.04
	L	-16,4,36	143	4.95
Dorsal pericentral gyrus	R	24,-26,72	86	4.64
Medulla	R	8,-28,-48	14	4.32
**Heart rate variability decreases (sd[pulse])**				
Anterior insula	L	-26,22,-8	80	4.33
Ventral occipital / lingual gyrus	R	24,-82,-4	55	4.23
Dorsomedial prefrontal cortex	L	2,28,44	69	4.05
**Arterial oxygen saturation increases (SpO**_**2**_ **)**				
No suprathreshold clusters	-	-	-	-
**Arterial oxygen saturation decreases (SpO**_**2**_ **)**				
Middle frontal gyrus	L	-48,36,26	57	5.21
Middle frontal gyrus	L	-38,34,24		4.08
**Minute ventilation increases**				
Thalamus	R	18,-12,6	106	8.85
Superior frontal gyrus	L	-28,24,46	389	8.16
Precuneus	L	-2,38,18	531	7.87
Lateral occipitoparietal junction	L	-42,-68,38	133	7.73
	R	50,-70,36	53	6.61
Insula	R	30,4,12	821	6.71
	L	-50,-4,16	164	5.59
	L	-34,-12,22	122	5.72
Inferotemporal cortex	L	-64,-14,-18	49	6.30
Cerebellum	L	-18,-46,-38	159	6.01
Orbitomedial cortex-frontal pole	-	-4,54,-12	160	5.33
**Minute ventilation decreases**				
Inferior occipital cortex	R	32,-96,0	437	4.70
	R	20,-58,2	121	4.33
	R	6,-74,2	76	4.01
Lateral occipital cortex	L	-38,-80,-6	303	4.60
Lateral occipitoparietal junction	R	28,-84,26	53	4.00
**Tidal volume increases**				
Mid insula	R	36,-6,6	5037	10.88
Precuneus	L	-4,-38,22	878	8.51
Anterolateral temporal cortex	L	-54,0,-10	734	7.39
Putamen	L	-20,-6,2	808	7.25
Superior temporal gyrus	L	-52,-32,6	574	7.07
Superior frontal gyrus	R	24,28,50	1373	6.88
Dorsal anterior cingulate	R	10,28,16		6.87
Central sulcus	R	44,-16,40	398	6.63
	L	-40,-16,40	46	5.274
Lateral orbitofrontal cortex	R	46,34,-8	210	6.54
Frontal operculum	L	-36,34,10	41	6.51
Superior frontal gyrus	R	24,50,24	102	6.05
Mid cingulate	R	4,-6,40	114	5.73
Hippocampus	L	-24,-18,-24	43	4.82
Lateral occipitoparietal junction	L	-40-74,86	46	4.77
Thalamus	R	14,-14,8		6.74
	L	-20,-12,12		6.59
Brainstem: dorsal pons		0,-26,-16	24	3.84
Brainstem: mid pons		0,-26,-32	9	4.03
Brainstem: medulla		2,-38,-44	33	5.31
**Tidal volume decreases**				
Head of caudate	R	10,24,2	6p	5.06
**Blood pressure increases (subset of 7 participants no voxel extent threshold applied)**				
Cerebellar vermis	-	0,-60,-28	22	8.65
Medulla	L	-6,-32,-48	23	8.10
Mid insula	R	50,0,2	6	7.72
Mid insula	L	-50,-8,2	8	6.16
**Blood pressure decreases**				
Premotor cortex	R	50,-4,30,	10	15.18
Middle frontal gyrus	L	-46,20,44	38	14.13
Inferotemporal cortex	R	46,-8,-28	33	12.04
Dorsal anterior cingulate	R	14,38,8	13	11.55
Inferior frontal gyrus	L	-62,14,22	24	9.71
Dorsal occipital cortex	L	-42,-64,26	22	9.67
Frontal operculum	L	-50,6,14,	10	9.35
Putamen	R	18,26,66	22	8.58
Temporoparietal junctions	R	48,-38,30	18	8.24
Mid insula	L	-32,-12,0	18	8.18
Mid insula	R	52,24,-2	15	8.07

#### Oxygen saturation

Oxygen saturation underpins the blood oxygenation level–dependent (BOLD) signal used in fMRI experiments to infer neural activity. The relative hypoxia induced in this experiment, as noted above evoked increases in activation across brain, rather than measurable diminution of the BOLD signal. Using arterial oxygen saturation (SaO2) as a continuous regressor for individual first level analyses resulted in no suprathreshold positively-correlated clusters of activity at group level, while activity within left dorsolateral prefrontal cortex increased with decreasing SaO2 (Table 2).

#### Respiratory rate

Activity within predominantly right dorsolateral prefrontal cortex and putamen correlated with increasing respiratory rate (across all conditions), while there was very little grey matter activity change (there was some periventricular signal change) associated with decreases respiratory rate. This lack of negatively-correlated response contrasts with the observed effects of task-condition described above (Table 2).

#### Minute ventilation and tidal volume

Voxel-wise activity showed much stronger association with changes in VE and VT. Activity increased within left dorsolateral prefrontal cortex, orbitomedial / frontal polar cortex, precuneus, bilateral mid insula and thalamus with increasing VE, which was also associated with decreases in activity within inferior occipital cortex. Activity within medulla, mid and dorsal pons, thalamus, bilateral mid dorsal insula, bilateral putamen and supragenual cingulate and right anterior insula tracked increasing VT, along with precuneus superior temporal gyrus and right parietal lobule. Head of caudate tracked decreasing VT (Table 2).

#### Heart rate and heart rate variability

Activity distributed across whole brain correlated with decreases in fluctuating heart rate. This encompassed focal activity changes in pons and thalamus, but was particularly reflected in the activation of bilateral caudate putamen and sensorimotor cortex. Anterior hippocampus, early visual cortex, temporal poles and cerebellum also showed this correlation with heart rate deceleration. There were no suprathreshold clusters of activity that correlated positively with heart rate increases.

Activity within the medulla and hippocampus correlated positively with continuous heart rate variability, derived here from inter-pulse interval variability (standard deviation over 15 seconds). In contrast, decreasing heart rate variability was associated with increased activity within anterior insula, dorsomedial prefrontal cortex (pre-SMA) and a region of left occipital cortex.

#### Blood pressure and blood pressure variability

Activity within medulla correlated positively with beat-to-beat blood pressure in the subset of participants in whom this was measured (N = 7). Correlated activity was also present within bilateral mid insula and cerebellar vermis. Regions including anterior insula/orbital operculum, insula, anterior cingulate, left dorsolateral prefrontal cortex and right temporoparietal junction showed an inverse relationship with beat-to-beat blood pressure fluctuations (Table 2).

#### Brainstem correlates of cardio-respiratory control

The brainstem mediation of cardio-respiratory changes was of particular interest to this study. As noted hypoxic challenge was associated with activity enhancement in dorsal mid pons, within a region encompassing the Kölliker-Fuse and parabrachial nuclei and locus coeruleus [13]. Blocks of slow paced breathing, compared to normal rate breathing, engaged this same region along with more rostral pontine regions bilaterally including the periaqueductal grey matter, into midbrain and hypothalamus (Fig 4). It is noteworthy that we did not observe brainstem activity reflecting the suprathreshold interaction between hypoxic challenge and paced slow breathing.

**Fig 5:**
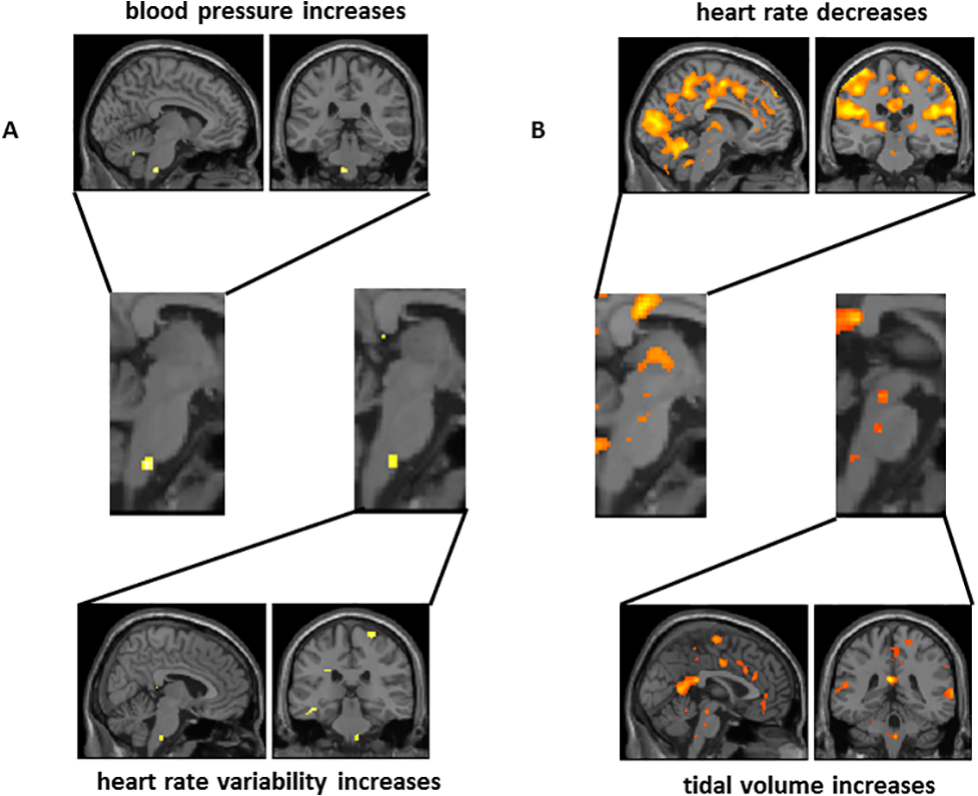
Focal brainstem activity correlating with physiological changes. Selected illustration of activity correlated with physiological changes. Data is illustrated at a voxel-wise threshold significance of P<0.001 uncorrected). **A)** within medulla, where the same locus reflects blood pressure increases and heart rate variability increases consistent with a neural substrate for the baroreflex mediating adjustments to hypoxic challenge. **B)** within pons and midbrain, where adjacent nuclei demonstrate relationships with decreases in heart rate and increases in tidal volume, putatively representing human homologues of brainstem centres supporting cardiorespiratory coupling as identified in experimental animals.

The correlations between brainstem activity and dynamic physiological changes across the experimental study provide further insight into proximal cardiorespiratory regulatory centres (Fig 5): Activity within ventral upper medulla tracked heart rate variability across all participants. The same region tracked beat-to-beat blood pressure fluctuations in those participants for whom these data were available, suggesting its direct participation in baroreflex control. Activity within the dorsal medulla correlated with changes in tidal volume, this region encompassing part of the nucleus of the solitary tract. Within the midbrain, PAG and a more anterior rostral midbrain region correlated with decreases heart rate over the course of the experiment. The latter centre overlapped with activity that increased with increasing tidal volume. Both heart rate decreases and increases in breath-by-breath tidal volume were also associated with activation of a mid pontine region immediately anterior to the region responsive to hypoxic challenges and paced slow-breathing (Fig 5).

#### Contribution of insular cortex

**Fig 6:**
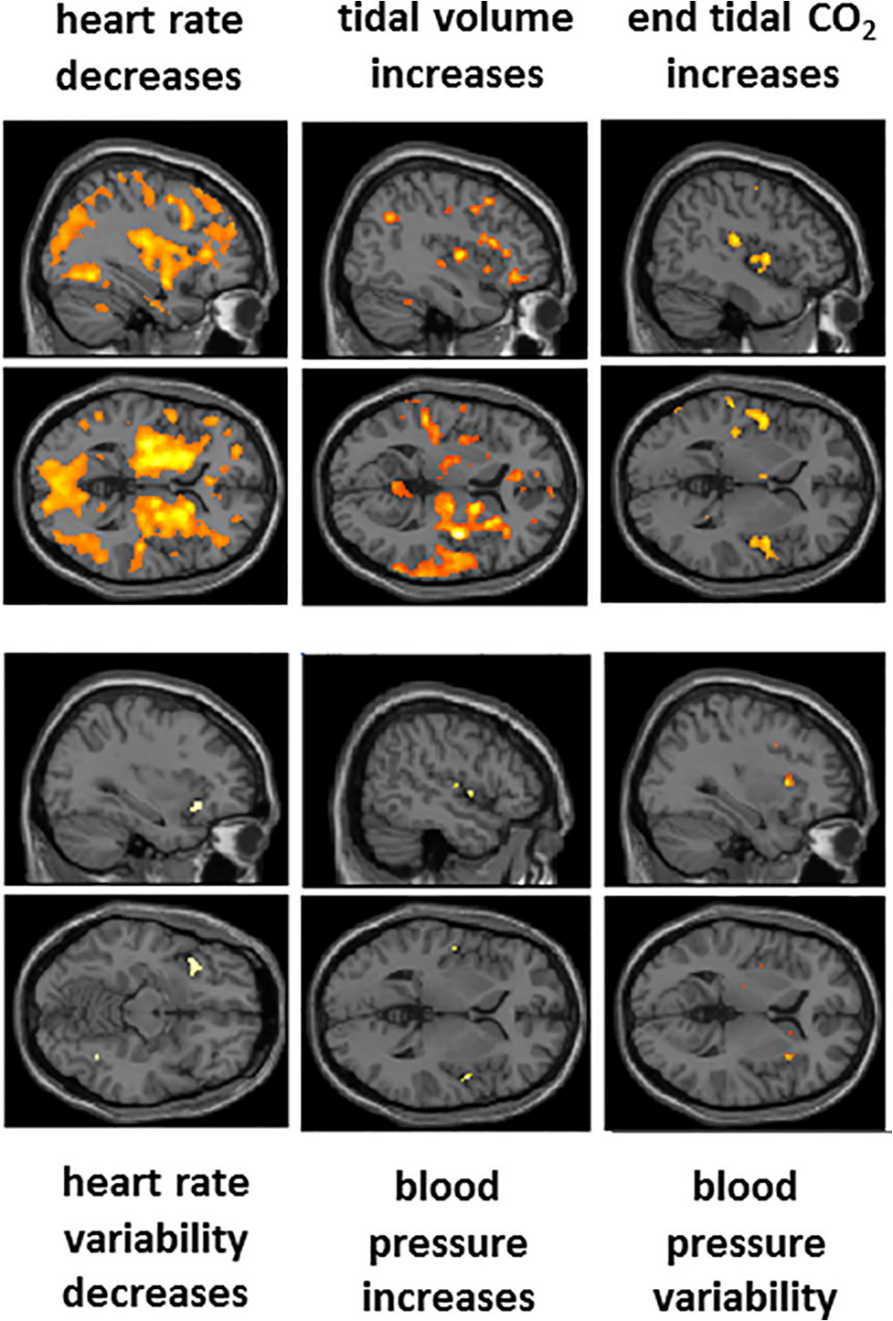
Focal activity within insula cortex correlating with physiological changes. Sagittal and horizontal sections from a standard template brain presented to highlight the location of activity changes within insula cortex associated with task-induced changes in physiological regressors. Of note are the deep insula components of signal change associated with heart rate decrease, merging with a marked engagement of basal ganglia, mirroring striatocortical activation previously observed in relation to expectancy-related heart rate deceleration [22]. The broadly distributed signal change, associated with this particular analysis arguably appears to carry most artefact, beyond what was controlled for by including movement, global and arterial O_2_ / end tidalCo_2_ regressors as confounding covariates within the analyses. Ventilation (VT) evoked predominantly right hemispheric changes in parietal and insula cortices and basal ganglia. Increases in end tidal CO_2_ was associated with enhanced activation within posterior ‘primary interoceptive’ insula, but did not impact global signal at threshold significance. Heart rate variability, blood pressure increases and blood pressure variability were associated with focal activation of distinct subregions of anterior and mid insula consistent with viscerotopography [23]. Data is illustrated at a significance of P<0.05 corrected, determined from the combination of voxel-wise significance and a cluster extent thresholds (see Methods).

The putative role of insula as viscerosensory cortex led us to specifically examine how activity within subregions [21] of insula reflected the experimental conditions and/or correlated with physiological changes during the experimental session. We retained the same significance threshold as for data presented elsewhere and did not apply less stringent local region-of-interest small volume corrections for statistical significance. Overall, the main effects of hypoxia or paced breathing at slow versus normal rates were not associated with marked changes in activation of insular regions. A discrete cluster of activity within left anterior (agranular) insula however did reflect interactions between these conditions. Across the experiment, much of the posterior-to-anterior extent of the insula, across granular, dysgranular to agranular regions bilaterally, showed enhanced activation with decreases in heart rate. This effect was also mirrored in enhanced activity within the adjacent basal ganglia. A similar pattern was seen in relation to increases in tidal volume, this time largely confined to right hemisphere and dominated by a discrete cluster of activity in posterior granular insula, with a further cluster in anterior agranular insula. Here also, subcortical caudate and thalamic activity was predominantly right-sided. Increases in end-tidal CO2 level evoked bilateral posterior / mid insula (granular/dysgranular) activity. Activity increases involving the same area in response to tidal volume change, suggests that this region may support the integrated representation of chemosensory and mechanoreceptive respiratory signals. It was therefore interesting that this granular insular region also reflected dynamic increases in blood pressure in the subset of participants for whom such data were acquired. Decreases in heart rate variability, likely reflecting baroreflex suppression, was associated with increased left anterior agranular insular activation, while increases in blood pressure variability (which in patient groups may represent a negative index of cardiovascular health) were associated with enhanced activation within right dorsal anterior (dysgranular) insular cortex (Fig 6).

## Discussion

Our study was driven initially by an interest in the reported health benefits and psychological consequences of slow breathing around six breaths per minute, which is observed to enhance baroreflex sensitivity and attenuate cardiovascular reactions to challenges such as hypoxia. Moreover, slow breathing training, in practices such as yoga, and in particular clinical groups, has reported subjective and clinical benefit. Here we applied a sophisticated combination of experimental respiratory physiology, detailed cardiovascular monitoring and functional brain imaging to characterise the neural mechanisms through which the central control of breathing interacts with autonomic cardiovascular responses, including those evoked during hypoxic challenge. Our findings provide novel insight into these mechanisms in humans, highlighting both brainstem and forebrain contributions to the integration of volitional breathing with internal bodily state, including adaptive cardiovascular responses. However, our study was insensitive to differential effects of slow breathing on subjective mood, likely attributable to (unnaturalistic) experimental procedures. However both our physiological and neuroimaging data do suggest mechanisms that might contribute to subjective psychological effects of slow breathing in other contexts (e.g. enhanced ventral striatal engagement when slow breathing during hypoxic challenge). This secondary question will be explored in further studies, which also exploit differences between trained ‘yogic’ breathers and individuals who have received no such training.

We tested the hypothesis that slow breathing modulates autonomic responses to hypoxia through a discrete set of neural processes. We show that these processes engender activity within brainstem centres supporting homeostatic reflexes, concomitantly with activity in forebrain regions, supporting volitional control, affective state and visceral sensation. These findings were derived from a sophisticated combination of functional neuroimaging, detailed physiological monitoring (including the capacity to explore brain correlates of beat-to-beat blood pressure) and interventional human experimental physiology. By identifying and dissociating regional patterns of brain responses to slow breathing, hypoxia, and their interaction, we provide insight into mechanisms associated more broadly with positive health benefits including an improved cardiovascular function and psychological wellbeing.

Neuroimaging studies of human physiology are benefitting from technical and methodological advances. For example, functional activation of brainstem nuclei can be confidently identified [24]. Different approaches exist to manage movement and physiological signals that have the potential to confound neuroimaging signals, particularly around brainstem [25] [26]. However, conventional methodological approaches, or modifications to these, complemented by concurrent physiological data acquisition, are able to achieve powerful results without *a priori* rejection of components of the data that may reflect functional interdependence of neural and other physiological signals [11] [24] [27–29]. In this context, the present study was undertaken with multiple physiological measures relating to respiration and cardiovascular function to provide interpretative insight to the neural responses accompanying the experimental manipulation of respiration. However, we also acknowledge limitation in the extent to which non-neural effects on BOLD signal can be controlled. By, providing a detailed account achieved uniquely through multiaxis physiological recording, our findings localize and quantify signal change that can represent artefact or physiological confound to conventional neuroimaging studies. A number of different technical approaches are available that aim to subtract physiological noise from functional neuroimaging datasets. There are diversely based on regression of global and tissue compartment signals or on decompositions informed by priors of cardiac and respiratory signal distributions [30] [31] and have reached widespread acceptance in the realm of resting-state studies. Some of the more sophisticated approaches in principle could have been applied also to the present task-related data; we employed only movement correction, proportional scaling of global signal and, for task conditions also inclusion of a SaO_2_ and CO_2_ regressors. However, because by definition physiological signals and neural representations of visceral state are at least partially correlated, over-correction would have confounded the results by removing variance associated with not only noise but also neural activity of interest. For the present study, we regard this as a more severe risk than that of false positives, since the risk of incorrect inference is on the whole mitigated by monitoring of multiple physiological axes and observation of convergent correlations within anatomical structures for which there is a priori evidence of involvement in homeostatic control.

Within brainstem, our study highlights the specific engagement of medullary and pontine nuclei in cardiorespiratory control, extending current knowledge regarding brainstem responses to respiratory challenge in animals and humans. Rodent studies point to a ventral respiratory column within pons and medulla containing inspiratory and expiratory premotor neurons and encompassing the preBötzinger nucleus which contains rhythmogenic neurons, particularly for inspiration. More rostrally, ventral to the facial nucleus, neurons within the retrotrapezoid nucleus drive expiration [8]. Interestingly, the brainstem activations observed within our study highlight dorsal nuclei more than this ventral column. While this observation is consistent with published human neuroimaging studies of respiration, where dorsal medullary nuclei (NA/NTS) and pontine centres show stronger activation to respiratory challenge, it is noteworthy that the ventral medullary nucleus we identify also reflects cardiovascular measures (increases beat-to-beat blood pressure and heart rate variability) which align closely to the baroreflex. Activity within a dorsal medullary region, encompassing NA/NTS, was enhanced by breath-by-breath increases in tidal volume. In contrast where we see activity within rostral ventral medulla, the association is with cardiovascular responses associated with baroreflex control (blood pressure and heart rate variability) rather than the respiratory challenges that ultimately engendered these cardiac responses. Activity within dorsal pons was also associated with increases in tidal volume, which was particularly apparent in the contrast between slow and normal paced breathing. The regions implicated, encompassing raphe and parabrachial nuclei, PAG and locus coeruleus, are contiguous with clusters of activity responding to hypoxic challenge (mid pons) and heart rate deceleration. Overall the picture within brainstem suggests functional modularity characterised by integrated cardiorespiratory control.

The observed pattern of brain response to hypoxic challenge fits with a stress reaction. Enhanced activity within bilateral amygdala, a region implicated in fear processing, is accompanied by engagement of ‘threat-sensing’ early visual cortices, ‘action-preparation’ pre-supplementary motor cortex, and the locus coeruleus region within pons (source of ascending noradrenergic ‘arousal’ projections). This constellation of affective neural responses could equally be elicited by a conditioned threat stimulus. In humans, knowledge regarding the functional changes of the brain to hypoxia is previously limited. In anaesthetised rodents, reduction of inspired O_2_ below 10% attenuates BOLD responses (i.e. the signal used to infer neural activity in fMRI experiments) [32]. In awake rodents, hypoxia increases cerebral blood flow, in part through effects of systemic cardiovascular responses [33]. In humans, cerebral blood flow increases with hypoxia, yet this may have inconsistent effects on effect on BOLD signal [34]. Our data are consistent with a coordinated neural response, heightening the overall BOLD signal across the brain and engaging brain regions typically linked to defensive and escape behaviours. We also observed hypoxia-related activity change within regions close to thalamus and potentially this may represent a homologue of the subthalamic region described by Reis and co-workers mediating hypoxia-related cerebral vasodilatation in conjunction with ventrolateral medulla [35].

Controlled slow breathing evoked widespread activity changes beyond the dorsal pons to engage hypothalamus and thalamus, basal ganglia, sensorimotor cortex and pre-supplementary motor area. Involvement of these latter motoric regions is seen also during breath holding and volitional hyperpnoea [12] [36–38]. The interaction between slow breathing and hypoxic challenge was one of the important motivators for the present study. Slow breathing at around six breaths per minute is associated with attenuated autonomic responses to hypoxic stress and preservation of baroreflex sensitivity in both healthy controls, individuals at altitude and in patients with heart failure and hypertension [3] [5] [39] [40]. Moreover there is a motivational element, since: the benefits of slow breathing are typically accompanied by subjective feelings of calmness, which potentially underlies the application of breathing control in meditation and yoga [6]. However, within our study, slow breathing increased tidal volume, but otherwise did not elicit major shifts in respiratory and autonomic parameters or subjective feelings state. A number of limiting factors are likely to account for this account for this divergence from earlier (laboratory) studies: the sample size was relatively low, and the participants (some of whom were ‘trained breathers’) had on average relatively slow spontaneous breathing rates. Also important was the degree of discomfort associated with the breathing apparatus within the constrained environment of the MRI scanner headcoil. Lastly, there was a trade-off between the duration of experimental periods optimal for ‘block-design’ fMRI studies and the duration of effective physiological manipulations. This led us to select brief periods of the different breathing conditions (1 minute duration i.e. 6 breaths for the slow paced breathing) which are much shorter than for published laboratory challenges. This certainly limited the magnitude of overall changes and, as such, there were no interactions between hypoxia and breathing rate expressed in peripheral physiological measures that attained criterion significance. Overall however, our confidence in the generalizability of findings, particularly within brain, is increased by the fact we observed some effects in the expected direction, despite the special conditions needed to undertake the combined physiological and neuroimaging study. It is also noteworthy that among the set of brain regions that did express significant interaction between hypoxia and breathing rate were bilateral ventral striatum, a region implicated in motivational drive, and the left frontal pole, arguably supporting psychophysiological and attentional regulation [41] [42]. Speculatively, this pattern of change may reflect a shift from the ‘distress state’ (in this case evoked by hypoxia) toward a more contemplative mental state enhancing attention and facilitating introspection.

Our observations for insular cortex provide partial insight into predicted emotional consequences of hypoxic challenge and its modulation by slow-breathing. Consistent with a viscerosensory role for insular cortex, activity changes accompanied alterations in heart rate, heart rate variability, respiratory volume, CO_2_ level, beat-to-beat blood pressure and short term variability in blood pressure. One surprise within these data is that activity was negatively correlated with heart rate in these experimental manipulations across bilateral insula, in association with similar changes throughout the neostriatum. This contrasts with a more usual pattern of insular activity associated with increases in cardiovascular arousal across neuroimaging studies. There were, nevertheless, posterior and mid insula responses to increases in blood pressure and left anterior insula responses to decreases in heart rate variability, both component indicators of baroreflex suppression [43]. Increasing end tidal CO_2_, another physiological driver of respiration, was also associated with posterior-mid insula activity enhancement. However, anterior agranular insular cortex, implicated in conscious access to viscerosensory representations and their translation into emotional feeling states, did not response robustly to hypercapnia or even hypoxic drive to respiration. These data, alongside evidence that panic may be elicited by respiratory stimulation independent of amygdala [44], may help inform targeted interventions for dyspnoea, its associated distress and expression in autonomic stress responses.

## Conclusions

The present study provides novel and detailed insights into human neurophysiological control. Brain responses to hypoxic challenge clearly relate to patterns of response observed to anxiety, stress and fear states induced by psychological as well as physical (e.g. pain) threat signals. The correlates of respiratory change, along mid and dorsal brainstem, thalamus and cortex and highlight the primacy of ventilation to the representation and motor control of breathing, particularly when engaged in controlled slow breathing. In contrast, activity within rostroventral medulla activity, a potential homologue of respiratory control centres identified in animals, was more closely associated with blood pressure and heart rate variability, putatively reflecting the influence of controlled breathing on baroreflex sensitivity. Particular insight is also gained regarding the role of insula, prefrontal cortex and associated basal ganglia in differential representation of interactions between volitional control of breathing and hypoxic challenge driving systemic physiological change in body. At a technical level this study demonstrates the value and feasibility of multiaxis physiological recording within an fMRI paradigm to enrich interpretative inference.
